# Autopsy practices for high-consequence infectious diseases: global guidelines, alternatives, and the BSL-4 gap

**DOI:** 10.1080/22221751.2026.2678656

**Published:** 2026-05-23

**Authors:** Kristina Allgoewer, Antonino Lavenia, Laura Secco, Tina Schaller, Antonia Fitzek, Charlotte Kriebel, Akhator Terence Azeke, Toshikazu Kondo, Rexson Tse, Paul Chui, Stefan Schmiedel, Benjamin Ondruschka

**Affiliations:** a Institute of Legal Medicine, University Medical Center Hamburg-Eppendorf, Hamburg, Germany; b Institute of Legal Medicine, University of Modena and Reggio Emilia, Modena, Italy; c Unit of Legal Medicine and Toxicology, Department of Cardiac, Thoracic, Vascular Sciences and Public Health, University of Padova, Padova, Italy; d Institute of Pathology and Molecular Diagnostics, University Medical Center Augsburg, Augsburg, Germany; e Center for Tropical Medicine, Bernhard Nocht Institute for Tropical Medicine & I. Department of Medicine, University Medical Center Hamburg-Eppendorf, Hamburg, Germany; f Department of Virology, Bernhard Nocht Institute for Tropical Medicine, Hamburg, Germany; g Department of Anatomic Pathology, Irrua Specialist Teaching Hospital, Irrua, Nigeria; h Department of Forensic Medicine, Wakayama Medical University, Wakayama, Japan; i School of Medicine and Dentistry, Griffith University, Gold Coast, Australia; j Health Sciences Authority, Singapore, Singapore

**Keywords:** Autopsy, forensic pathology, viral haemorrhagic fevers, biosafety level 4, post-mortem procedures, high-consequence infectious diseases

## Abstract

Autopsies play a critical role in elucidating the pathogenesis of emerging infectious diseases, particularly in cases involving high-consequence pathogens such as viral haemorrhagic fevers (VHFs). While biosafety concerns have restricted post-mortem examinations in such contexts, the COVID-19 pandemic has renewed interest in autopsy-based research and highlighted both the potential and the gaps in current biosafety protocols. This narrative review outlines autopsy practices in the context of high-consequence infectious diseases (HCIDs) with a focus on VHFs, summarizes reported autopsy cases, explores alternative post-mortem methods, and examines the evolution of legal and institutional frameworks in response to the pandemic. A comparison of official international guidelines shows that while detailed autopsy protocols have been published for pathogens requiring BSL-3 containment – particularly in the United States (US) and United Kingdom (UK) – no official procedural guidance seems to be available for performing autopsies under BSL-4 conditions. Instead, current recommendations at this level are limited to post-mortem handling and disposal of the deceased. This regulatory and procedural gap underscores the urgent need for harmonized, high-containment autopsy protocols that balance biosafety with scientific value. Developing such frameworks will be essential to improve outbreak preparedness and enable evidence-based responses to future pandemics globally. Accordingly, we propose a structured, system-based approach to BSL-4 autopsy practice as a foundation for discussion and future guideline development.

## Introduction

Despite a significant decline in the number of autopsies performed over recent decades, post-mortem examinations remain irreplaceable for determining the real cause of death as well as for providing critical insights into underlying pathophysiological mechanisms, which form the basis for developing effective therapeutics and vaccines [1–4]. Their significance becomes even more pronounced in the context of high-consequence infectious diseases (HCIDs), which are defined as acute human infectious diseases marked by high morbidity and mortality, a lack of effective therapeutic or preventive interventions, and significant potential for transmission in community and healthcare settings.

Most diseases on the evolving list of HCIDs are caused by pathogens classified in the highest risk group (RG4), which typically require the highest level of biocontainment (biosafety level 4, BSL-4) [5]. These pathogens include viral haemorrhagic fever (VHF) viruses of zoonotic origin such as Ebola virus, Marburg virus, Crimean-Congo haemorrhagic fever virus, and Lassa virus, which are primarily transmitted through contact with an infected host (e.g. rodents, bats) or via arthropod vectors. In recent decades, several outbreaks of high-consequence VHFs have occurred, and in some regions – particularly in sub-Saharan Africa and parts of South America and Asia – these diseases remain endemic and continue to pose a persistent public health threat. However, countries in temperate climates, including Europe, are not immune to risk. Sporadic imported cases [6–8] have underscored the need for robust response systems to prevent potential epidemics as well as for the development of specific therapeutics – an urgency that is likely to grow amid increasing global mobility and climate-driven changes in disease ecology [9].

Human-to-human transmission can occur through direct or indirect contact with infectious bodily fluids such as blood, vomit, urine, or faeces. Additionally, airborne HCIDs such as Middle East Respiratory Syndrome (MERS), Severe Acute Respiratory Syndrome (SARS), and Nipah virus can spread via respiratory droplets or aerosols [10]. In healthcare or laboratory settings, these transmission routes can facilitate nosocomial spread, especially where infection prevention and control (IPC) measures – such as patient isolation and appropriate use of personal protective equipment (PPE) – are non-existent or insufficient [11,12].

This transmission risk is particularly relevant in the context of post-mortem examinations, where contact with infectious tissues and bodily fluids during or after autopsy practices poses a considerable risk to personnel, especially in the absence of appropriate biosafety measures [13]. In primates, Ebola virus has been found to be viable for up to 7 days after death, and a documented imported Lassa fever case in Germany involved transmission to a funeral director who handled the corpse [8,14]. Although numerous protocols describe in detail the biosafety measures applied to prevent transmission in laboratory and healthcare settings, detailed and authoritative guidelines for conducting autopsies under BSL-4 conditions are, to date, still lacking.

While VHFs are projected to cause 26,000 deaths each year, autopsies on high-risk cases are extremely rare [15]. As a result, this limitation hampers the study of VHF pathophysiology and, consequently, the development of specific therapeutics, thereby contributing to continued reliance on supportive care or broad-spectrum antivirals with questionable effectiveness [16]. The available evidence on autopsies in cases of VHF is largely limited to isolated case reports and small case series, which are summarized in the following section.

In this narrative review, we aim to provide a comprehensive overview of autopsy practices and biosafety considerations in the context of HCIDs, with a particular focus on VHFs. We provide an overview of reported autopsies in cases of VHF, examine existing regulations and legal frameworks governing HCID autopsies, and reflect on biosafety lessons learned during the COVID-19 pandemic. We also discuss possible alternatives to conventional autopsies in high-risk cases and compare current official guidelines for conducting autopsies under BSL-3 conditions as well as available protocols for BSL-4 post-mortem handling and care. In addition, we outline a structured, system-based approach to BSL-4 autopsy practice intended to inform discussion and future guideline development. This review is based on a targeted literature search of PubMed and official institutional websites up to August 2025. Only official, publicly available guidelines were included for the comparison; single-centre protocols and informal practice reports were excluded. No systematic review protocol was applied.

## Documented autopsies in cases of viral haemorrhagic fever

While some HCIDs, such as MERS, SARS, and Mpox, may be managed under BSL-3 or enhanced BSL-3 conditions depending on national guidelines and clinical context, viral haemorrhagic fevers caused by RG4 pathogens – such as Ebola virus, Marburg virus, and Lassa virus – require BSL-4 containment. In confirmed cases, autopsies have been performed only under exceptional circumstances – typically when the risk-benefit assessment clearly favoured the procedure, or when the diagnosis had not been recognized prior [17,18].

The documented autopsies in cases of VHF requiring BSL-4 containment illustrate both the risks and the scientific value of post-mortem examination: In 1970, several autopsies on deceased Lassa fever patients were performed in Nigeria to investigate the newly identified virus. Tragically, during one of the procedures, a physician reportedly sustained an accidental cut and later died from the disease, underscoring the substantial hazards associated with autopsies involving RG4 pathogens and the need for stringent biosafety measures [19,20]. Despite this incident, another autopsy study on Lassa fever was published in 1982 and remains a key reference to this day [21]. Conducted as part of a field investigation in Sierra Leone, it included post-mortem examinations of 21 virologically confirmed Lassa fever cases, including six complete autopsies. The study documented consistent necrotic lesions in the liver, adrenal glands, and spleen, along with high viral loads in multiple organs including liver, lung, spleen, kidney, heart, placenta, and mammary gland. Notably, the pathological findings were not considered sufficient to explain the fatal course of the disease, suggesting that immunopathological mechanisms may play a critical role in disease progression. Despite being more than four decades old, this study continues to provide valuable insights into the organ tropism and systemic effects of Lassa virus infection – and illustrates the unique potential of BSL-4 autopsies to advance our understanding of the pathogenesis of high-consequence viral diseases. Strikingly, the article provides no information on any biosafety precautions or personal protective measures, highlighting how little attention was given to such aspects at the time.

Investigations of Ebola and Marburg virus infection further exemplify the dual role of post-mortem examinations as both a source of occupational risk and a critical tool for advancing scientific understanding. The first identification of Taï Forest ebolavirus (formerly Côte d’Ivoire Ebola virus) was linked to a human infection acquired during the autopsy of a wild chimpanzee in 1995 in Côte d'Ivoire [22]. During the first recorded Marburg virus outbreak in 1967, a pathology technician sustained a knife injury to the forearm during a post-mortem examination and subsequently developed secondary infection [23]. Yet, a 1998 case study of a fatal Marburg virus infection in a 15-year-old European male who had been visiting Kenya employed immunohistochemistry and electron microscopy on autopsy tissue to map viral distribution and associated tissue damage – analyses that were only made possible through a full post-mortem examination [24]. The study revealed a previously unreported tropism for pancreatic islet cells and confirmed macrophages, hepatocytes, and adrenal cells as key targets of infection, underscoring the value of high-resolution imaging in both diagnosis and pathogenesis research of filoviral disease. While the authors emphasize the need for rapid and specific diagnosis to enable appropriate treatment and protect healthcare workers, they similarly provide no details on biosafety precautions or personal protective measures used during the autopsy.

Although dengue virus infection is not classified as a HCID, large-scale autopsy studies conducted during dengue outbreaks illustrate the insights that systematic post-mortem investigations can provide into VHF pathophysiology [25–30]. Published case series and surveillance-based autopsy studies from endemic regions have identified organ-specific pathology, cellular targets of infection, and previously unrecognized fatal cases, thereby improving understanding of disease mechanisms and burden. These findings underscore the scientific value of structured autopsy programmes for VHFs and highlight the knowledge gap that persists for VHFs caused by RG4 pathogens, where comparable post-mortem data remain largely unavailable [31].

## Autopsy alternatives in high-risk and resource-limited settings

In the absence of BSL-4 autopsy guidelines, alternatives to conventional autopsies have emerged in recent years, particularly in settings where biosafety concerns, cultural factors, or logistical constraints limit the feasibility of full post-mortem examinations – most notably post-mortem imaging as a virtual autopsy (Virtopsy) [32] and its procedural extension, minimally invasive tissue sampling (MITS) [33,34]. Virtopsy relies exclusively on imaging techniques – such as computed tomography (CT) and magnetic resonance imaging (MRI) – to examine the body post-mortem. While this approach significantly reduces the risk of infection and enables the identification of indirect pathological signs (e.g. haemorrhages, body cavity effusions, inflammation), the applicability of CT- and MRI-based virtopsy is often limited in low-income settings due to the high costs and restricted availability of advanced imaging infrastructure. Moreover, it typically does not permit the collection of biological specimens for histopathological or microbiological analysis at all.

In contrast, MITS is a needle-based post-mortem technique that combines imaging modalities with targeted biopsies to obtain tissue samples from major organs as well as bodily fluids. Initially developed and applied in low- and middle-income countries, MITS is frequently performed using ultrasound guidance or, where imaging is unavailable, through standardized anatomical landmark-based approaches. However, MITS has also regained global relevance during the COVID-19 pandemic, when infection control concerns and logistical constraints renewed interest in minimally invasive post-mortem approaches [35,36].

An advanced extension of the MITS concept is robotic post-mortem biopsy (RPMB), which combines CT-based planning with robotic needle insertion to obtain tissue samples without direct contact with the body. Designed to enhance biosafety in high-risk contexts, the RPMB system enables safe and accurate sampling of internal organs from corpses enclosed in protective body bags, thereby reducing infection risks for personnel. The setup, based on a lightweight, mobile robotic arm and an open-source planning module, allows for flexible deployment and integration into post-mortem imaging workflows. In a virtual reality setup with a digital twin, fully remote planning and control of robotic post-mortem biopsies may be realized. By avoiding direct exposure and enabling precise sampling, RPMB exemplifies how robotic systems may expand the toolkit for MITS approaches under high-containment conditions [37,38].

Although these techniques offer a safer and less invasive alternative to full autopsy, they are subject to notable limitations. These include the limited availability of imaging and biopsy equipment, as well as intrinsic procedural constraints: tissue samples are often small, selectively obtained, and not comprehensive, increasing the risk of missing focal or subtle lesions (e.g. nodules, abscesses, or areas of organ ischaemia) and neglecting relevant organ tissues such as the brain [39–42]. These limitations are widely recognized and continue to represent a significant barrier to the full replacement of conventional autopsy.

While these alternatives offer valuable tools in resource-constrained settings, they cannot fully compensate for the lack of comprehensive autopsy data in high-consequence infection scenarios. Yet the decision to perform such procedures is often shaped by legal and institutional frameworks. The following section examines how these frameworks have evolved in the wake of the COVID-19 pandemic. Importantly, COVID-19 was classified as a HCID during the early stages of the pandemic, illustrating both the evolving nature of the HCID concept and the need for adaptable regulatory and biosafety frameworks during emerging outbreaks [5].

## Evolving regulation of infectious disease autopsies in the post-COVID era

More than a decade prior to the COVID-19 pandemic, a cross-sectional survey conducted across 48 isolation facilities in 16 European countries revealed substantial gaps in preparedness for the post-mortem management of HCID cases. Only 8.3% of facilities reported having all key safety features in place – including written procedures, trained personnel, appropriate infrastructure (such as BSL-3 autopsy rooms), and specific protective equipment – while over 10% lacked even the most basic provisions [43].

The COVID-19 pandemic vividly demonstrated the importance of autopsies for uncovering the pathophysiological mechanisms of novel viral pathogens. As a result, post-mortem examination regained visibility and acceptance as a key investigative tool in infectious disease research not only within the research community but also among the general public [3,44]. One of the most prominent national responses to this renewed interest in autopsy-based research emerged in Germany, where institutions began collaborating within the first weeks of the pandemic. By the end of 2020, a dedicated national autopsy network (NATON) was established, with a centralized registry serving as its digital backbone [45]. Bringing together the disciplines of pathology, forensic pathology, and neuropathology for the very first time, the network has enabled numerous multicentre research projects based on post-mortem tissue samples and contributed to the development of a German S1 (consensus-based) guideline for clinical autopsies [46].

Notably, this progress unfolded in spite of early official recommendations in Germany that discouraged autopsies due to biosafety concerns. The Robert Koch Institute (RKI), Germany’s national public health institute responsible for disease control and prevention, initially issued recommendations discouraging autopsies due to concerns about potential infection risks [44,47]. However, in response to objections from German researchers and forensic pathologists emphasizing the scientific value of autopsies, the RKI revised its guidance, ultimately contributing to a reversal of the initial cautious stance [46,48]. In 2021, following intensive dialogues with the Federal Ministry of Health, NATON played a key role in initiating amendments to the German Infection Protection Act (IfSG §§ 25, 60), aimed at increasing the number of autopsies mandated by local health authorities in the context of infectious disease outbreaks and for general population safety reasons.

The pandemic also prompted professional societies as well as individual medical centres and forensic institutes around the world to develop their own recommendations for the safe conduct of autopsies in infectious disease cases. These protocols – typically designed for BSL-2 or BSL-3 conditions – addressed both research-oriented and forensic post-mortem procedures. Published case reports and small case series describe the use of enhanced PPE, including respirators or powered air-purifying respirators (PAPRs), as well as procedural adaptations such as the use of handsaws instead of oscillating saws, the instillation of formalin into the mouth, nose, and pharynx of the corpse two hours prior to autopsy, and in-corpore dissection techniques [49–55].

Beyond written protocols, some institutions also introduced concrete infrastructural innovations to address specific biosafety challenges during autopsies. For instance, forensic pathologists in Malaysia developed a custom-built craniotomy box to contain aerosols during skull opening procedures on deceased COVID-19 patients. This transparent, barrier-like enclosure was successfully tested in a BSL-3 autopsy suite and confirmed via surface swab real-time reverse transcription PCR to prevent viral dispersion to the box outside during the procedure [56].

While the COVID-19 pandemic has renewed interest in autopsies as a tool to explore the pathophysiology of infectious diseases, this has not yet translated into the establishment of guidelines for BSL-4 autopsies. Since BSL-4 agents are associated with markedly higher mortality rates and, in most cases, lack approved treatments or vaccines, any infection must be prevented wherever possible, rendering residual risks unacceptable at the BSL-3 level.

## Managing the deceased in BSL-4 contexts: post-mortem care guidelines compared

A review of current official BSL-4 guidelines on handling the deceased – including those from the U.S. Centers for Disease Control and Prevention (CDC, USA) [57,58], the World Health Organization (WHO) [59–61], RKI (Germany) [62–64], the UK Department of Health and Social Care [65–67], and the Australian Department of Health, Government and Ageing [68] – shows that full autopsies in BSL-4 cases are either strongly discouraged or allowed only in exceptional circumstances. Some documents explicitly recommend less invasive alternatives, such as targeted biopsies, for high-risk cases, and none of the guidelines provide comprehensive protocols for conducting complete BSL-4 autopsies. The Australian guidance, for example, allows coroner-directed autopsies following consultation with public health units and advises applying the precautions used for surgical procedures on Ebola patients, yet a detailed protocol for these procedures is not provided. Overall, official documents focus on post-mortem handling, transport, and disposal (Supplemental Table S1), leaving a notable procedural gap in high-containment autopsy practice.

A comparison of those BSL-4 post-mortem care guidelines issued reveals a general consensus on biosafety measures in the handling of the deceased, with broad agreement on PPE use, body containment, and funeral restrictions, but differences in scope, level of procedural detail, and the explicitness with which certain steps are addressed. While the Australian guidance is restricted to Ebola, the WHO and German guidelines address both Ebola and Marburg viruses. The CDC further extends its scope to include Lassa fever, Crimean-Congo haemorrhagic fever, and South American VHFs, whereas the UK guidance encompasses literally “all” VHFs. Although Rwanda’s Ministry of Health has issued general recommendations for the safe and dignified burial of patients who died from Marburg virus infection [69], these documents lack sufficient detail on post-mortem care to allow meaningful comparison with the more comprehensive national and international guidelines and are therefore excluded. The 2024 Marburg outbreak was the first reported in Rwanda; with 66 confirmed cases and 15 fatalities, it ranked among the largest documented worldwide, yet no autopsies of confirmed cases were reported [70].

Across the reviewed guidelines, PPE ensembles show substantial overlap with some notable differences. Most do not define post-mortem-specific sets but refer to those used in clinical VHF/HCID care, with minor additions such as aprons in Australia. All require respiratory, head, and face protection, though the specified level differs: CDC, WHO, and Australia accept N95/FFP2 respirators with goggles or a face shield and a hood, or PAPRs, whereas Germany and the UK mandate at least FFP3 (≈N99), with the UK additionally requiring full hoods. While sustained airborne transmission of Ebola and most VHFs is unproven, aerosol-generating procedures justify the recommendation for high-level respiratory protection.

All guidelines call for layered gloves, impermeable gowns or suits, and protective footwear, but differ in disposability versus durability. CDC/WHO/Australia accept double disposable gloves, gowns, and aprons, with added mention of puncture-resistant soles; Australia discourages coveralls due to risk of heat stress and contamination during doffing. Germany and the UK mandate triple gloves, reinforced suits, and heavy-duty boots, emphasizing cut resistance. The key contrast is thus between lighter, disposable ensembles designed for practicality in outbreak settings and more elaborate configurations prioritizing maximum durability and protection against sharps injuries.

For funeral and ritual practices, all guidelines emphasize the need to minimize physical interaction with the deceased. Washing and embalming are uniformly prohibited and viewing or direct contact is generally restricted. The main differences concern how cultural and familial needs are addressed. The UK guidance explicitly states that while the wishes of relatives should be respected as far as reasonably practicable, the public health risks necessitate strict limitations. WHO guidance goes further in embedding cultural and religious considerations. In community settings, it provides protocols for dignified burials in both Christian and Muslim traditions and requires burial teams to include a communicator and a religious representative. Teams are instructed to offer condolences while also encouraging family members to perform communal hand hygiene after the funeral.

## Procedural guidance for BSL-3 autopsies: comparative insights

Although comprehensive procedural guidance for full autopsies under BSL-4 conditions remains absent, authorities in the US and the UK have developed official autopsy protocols for infectious diseases requiring BSL-3 containment. A comparative review of published BSL-3 autopsy guidelines (Table 1, columns 1–3) issued by the CDC [71,72] and the UK’s Royal College of Pathologists (RCPath) [73] shows that both address core domains but differ markedly in scope, level of detail, and presentation.

**Table 1.** Comparison of published BSL-3 autopsy guidelines and suggested approach for BSL-4 Guidelines.^a^

Aspect	USA (CDC)		UK (RCPath)	Our Approach
Biosafety level	BSL-3			BSL-4
Scope	COVID-19 [71]	Mpox [72]	HG3 agents^b^ [73,74]	RG-4 pathogens
Autopsy indication/Risk assessment	Necessity to be determined by professional judgement (taking into account medicolegal jurisdiction, facility environmental controls, PPE availability, and family and cultural wishes); site-specific risk assessment prior to conducting any procedures	Autopsies only to the extent required to obtain needed information; omit examinations that generate aerosols and increase risk of environmental contamination	Risk assessment of each case required; may include clinical history, pathological information, infection control information, external examination of body; MITS sufficient for systemic infections when sampling blood, liver, and spleen; ensure mortuary facilities and personnel expertise to be sufficient for that infection	Subject to applicable legal or public health requirements; case-specific risk assessment required (including available clinical and epidemiological information, infection control considerations, and external examination); invasive procedures may be performed when justified by diagnostic, public health, or substantial scientific research objectives and authorized by the competent authority, provided that institutional and operational prerequisites for BSL-4 autopsy practice are met
Facility	**Separation:** Airborne infection isolation rooms (if available); precautionary signage on entry door; doors kept closed except during entry and egress	**Separation:** Autopsy suite (not specified); doors and windows kept closed during autopsy	**Separation:** Separate high-risk room recommended (not mandatory), at the minimum adequate space away from other activities	**Separation:** Autopsy procedures restricted to full BSL-4 containment within a physically separate high-level isolation unit or a dedicated BSL-4 autopsy facility; mandatory spatial separation of the high-risk autopsy area, with appropriate ante- and transition chambers separating it from adjacent zones
	**Air Handling:** ≥12 air-exchanges per hour (≥6 for older structures), negative pressure relative to adjacent passageways and office spaces, outdoor air exhaustion (away from human traffic/gathering/other air intake systems), local airflow control (laminar flow systems)	**Air Handling:** ≥12 air-exchanges per hour, negative pressure relative to adjacent passageways and office spaces, outdoor air exhaustion (away from human traffic/gathering/other air intake systems), local airflow control (laminar flow systems)	**Air Handling:** Whole-room ventilation systems with downward airflow from ceiling to floor; alternatively, down-draft tables	**Air Handling:** Dedicated BSL-4 ventilation system with inward directional airflow, sustained negative pressure relative to adjacent areas, and unidirectional air movement; HEPA-filtered supply and exhaust air, with exhaust discharged directly outdoors to unoccupied areas via a sealed system; controlled pressure gradients with interlocked doors between zones; continuous monitoring of ventilation and pressure systems during operations
	**Additional Regulations:** Biosafety cabinet (class II or higher); work surfaces with integral waste containment and drainage features; written biosafety policies, site-specific risk assessments, and procedures	**Additional Regulations:** Biosafety cabinets (not specified)	**Additional Regulations:** Appropriate sampling technical systems and access to appropriate microbiology laboratory facilities; all necessary equipment in room; generate (and update) SOPs for managing specific high-risk infectious autopsies and to cover all autopsy scenarios (common and uncommon)	**Additional Regulations:** Facility formally designated and licensed for BSL-4 operation; validated and regularly tested containment systems; comprehensive, facility-specific SOPs defining requirements for all stages of operation, including autopsy procedures, waste and wastewater management, emergency and failure scenarios (e.g. man-down, system failure), and coordination with external emergency services; established provisions for full facility decontamination prior to maintenance or repair; controlled access, documented oversight by competent authorities, and continuous technical monitoring during operations
Staff	Team limited to minimum number of people necessary for safely conducting autopsies, logbook of all names, dates, and activities (including custodial staff)	Not specified	Team including pathologist, anatomical pathology technologist as well as circulator assistant (not mandatory); prior to procedure, ensure technologists are comfortable proceeding; ensure staff has appropriate vaccinations and is in agreement of protocols	Team limited to the minimum number of personnel required for safe operation; participation restricted to specifically trained staff with documented competence in PPE use and informed agreement to perform BSL-4 procedures; clearly defined functional roles, including autopsy procedures, operational oversight and safety monitoring, logistical support and material transfer, decontamination functions, and technical or engineering support; independent oversight of procedural compliance and system performance; emergency response protocols in place; vaccination provided where available
Training/Supervision	Infection control and biosafety training mandatory; participating personnel trained in policies, procedures, PPE handling	Not specified	All staff must be aware and in agreement with protocols to manage infections; pathology trainees must be introduced to safe practice on cadavers with HG3 infection, may undertake high-risk infection autopsy work under consultant supervision	Mandatory infection control and biosafety training; initial and ongoing competency-based training in BSL-4 policies, procedures, and PPE use; comprehensive awareness of and compliance with facility-specific SOPs at all stages of operation; participation subject to regular refresher training and simulation-based drills; real-time supervision and documented monitoring of procedures, with oversight by experienced BSL-4 personnel and biosafety or infection control experts
PPE	Face shields or goggles covering front and sides of the face; N95 respirators or higher, PAPR recommended due to high likelihood of aerosol generation; double surgical gloves with an interjacent layer of cut-proof synthetic mesh gloves; surgical scrub suits and caps, impervious gowns or aprons with full sleeve coverage, shoe covers with non-slip tread; specific standardized don, use and doff protocols (e.g. EPA-compliant disinfection prior to removal, cleaning and disinfection of reusable PPE, use of appropriate laundry or waste receptacle)^c^		Clear visor protecting face, eyes, and mouth; surgical mask or FFP3 (case-dependent), hair protection, surgical scrub suit, whole-body waterproof gown (including forearms), plastic apron, latex (or equivalent) gloves with protective/cut resistant under-gloves made of Kevlar or neoprene; potentially metal fine mesh gloves (for sawing); rubber boots with metal-protected toecaps/dorsal reinforcement	BSL-4 standard PPE; fully encapsulating positive-pressure suits with dedicated, HEPA-filtered breathing air for personnel performing high-risk autopsy procedures; role-adapted PPE for supporting functions, including material handling, logistics, and decontamination, typically comprising PAPR-based respiratory protection and fully protective suits; multiple layers of gloves, including cut-resistant protection where indicated by task; integrated protective footwear; mandatory, validated donning, doffing, and chemical decontamination procedures for all PPE and personnel prior to exit from containment
Injury prevention	Cautious handling of needles and sharps (e.g. no recapping, bending, or cutting); disposal in appropriate sharps containers		Use of round-ended scissors and PM40 blades with blunted points, minimal use of sharps; no resheathing of needles, disposal in sharps buckets; operate within body cavity one at a time	Strict minimization of sharps use; preference for safety-optimized and blunt-ended instruments where feasible; cautious handling of needles and sharps (e.g. no recapping, bending, or cutting) with immediate disposal in approved sharps containers; procedural strategies to limit exposure, including controlled and sequential work within defined body cavities; validated decontamination or sterilization of instruments following use
Saw use	Oscillating bone saw should be avoided, only permitted with vacuum shrouds; hand shears as an alternative cutting tool should be considered	Oscillating bone saw should be avoided, only permitted with vacuum shrouds	Oscillating saw use permitted with vacuum evacuation into separate chamber (air extraction hood) or hand saw (with chainmail glove); for (suspected) TSE: encase head, operator’s hands and saw in transparent plastic bag	Use of oscillating bone saws permitted within full BSL-4 containment when justified by procedural requirements and supported by appropriate engineering controls; preference for techniques that minimize aerosol generation; bone cutting procedures performed under dedicated local exhaust ventilation or equivalent containment measures; procedural pauses implemented as appropriate to allow clearance of airborne contaminants; additional physical encasing measures not routinely required under full BSL-4 conditions
Post-mortem specimen for diagnosis	Multiple respiratory tract swabs (e.g. nasopharyngeal swab, lung swab from each lung)	Not specified	Case-dependent: may include blood, urine, cerebrospinal fluid, nasal swabs, dab cytology of lesions (bacterial and fungal infections), brain smears (diagnosis or exclusion of cerebral falciparum malaria)	Specimen collection limited to those required for diagnostic or approved research purposes; all specimens handled and packaged within BSL-4 containment; validated multi-step decontamination and secure, leak-proof packaging prior to any transfer; clear biosafety hazard labelling of specimen containers; laboratories receiving specimens informed in advance; specimens released from containment only following validated inactivation procedures or transfer under approved high-containment transport by authorized providers
Autopsy tissue samples	Lung (≥3 samples, preferably from different locations and any areas of lesions), airway (≥1 section including trachea, bronchi or both airways), other major organs if involved as suggested by clinical history/laboratory findings or to evaluate possible extrapulmonary complications; approximately 5 mm in thickness; fixed in 10% buffered formalin for 72 h	Multiple tissue samples from all major organs (e.g. skin, spleen, lymph nodes, liver, lung, kidney, heart, GI tract, adrenals, major reproductive organs, brain, bone marrow); fixed in 10% buffered formalin for ≥48 h and ≤2 weeks for all organs except brain (≤4 weeks), then paraffin-embedded; second set: sterile 1.5–2 mL screw-capped plastic vials with O-ring or any leak-proof tube, without any viral transport media addition, stored and shipped frozen at −20°C or lower	Multiple tissue samples from various organs depending on confirmed or suspected infection and whether local or systemic, fixed in formalin for >48 h (for TSE: subsequent treatment with 96% formic acid); systemic infections: obvious lesions plus standard sample set comprising lungs, heart, spleen, liver, largest lymph node, lumbosacral bone marrow, kidneys (adrenal, gut, brain, skin, bladder, and genital tract recommended); local infections: tissue-specific samples (e.g. brain for suspected encephalitis)	Multiple tissue samples from organs selected according to confirmed or suspected infection; standard formalin fixation for histological samples for ≥48 h; systemic infections: lung, heart, spleen, liver, lymph nodes, lumbosacral bone marrow, kidneys; adrenal glands, GI tract, brain, skin, bladder, and genital tract (as appropriate); localized infections: tissue-specific sampling (e.g. brain for suspected encephalitis); native (non-formalin-fixed) tissue sampling permitted where justified by diagnostic or approved research requirements, retained within BSL-4 containment or released only following validated inactivation and handling protocols
Post-mortem containment	Body bagging with single or double body bag of ≥152 µm thickness, external disinfection of bag	Prior to autopsy: body wrapping in plastic shrouds; placement of wrapped corpses on mortuary stretchers with clean linen sheets for morgue transport, body bagging for mortuary transfer	Number of necessary body bags (0, 1, or 2) specified depending on pathogen; use leak-proof bags and wadding around body if necessary; if brain removed and not replaced, wadding in cranial cavity	Multiple leak-proof body bags (≥2) with sequential external decontamination between containment layers, according to pathogen-specific validated protocols; secure containment and return of organs and tissues to the body using leak-proof packaging prior to closure; use of absorbent wadding as required to prevent leakage (including cranial cavity if opened); final placement in securely sealable and lockable coffin for transport and burial
Cleaning/Exit protocol	Active ventilation systems and PPE (gloves, eye protection, clean, long-sleeved fluid-resistant gown and N95 or higher) during cleaning; surface-specific disinfectants; adsorbent materials and tongs/other utensils for gross contamination and liquids collection; avoidance of compressed air and/or pressurized water; incineration of sharps containers; cleaning and disinfection or autoclaving of non-disposable instruments; leak-proof biohazard bags for laundering contaminated textiles; removal of PPE followed immediately by washing of hands following CDC hand hygiene recommendations	Cleaning and disinfection of all surfaces using 0.5% sodium hypochlorite/EPA-registered disinfectants, cleaning and disinfection of all reusable equipment; use of biohazard bags for non-reusable specimens and barrier protection materials collection; removal of PPE followed immediately by washing of hands with water and soap for 20 s (if not available, use of 60–95% alcohol-based sanitizer as alternative)	Surfaces and equipment disinfection and cleaning, non-disposable instruments decontamination, appropriate specimen containers; removal of any PPE and contaminated clothing; disposal of single-use PPE in the correct waste stream in dedicated labelled containers; placement of all reusable infectious/contaminated work clothing directly in a sealed water-soluble/alginate bag inside a plastic bag before placing it in a laundry receptacle; clean hands with soap and warm water	Cleaning and decontamination performed according to validated BSL-4 facility protocols; chemical, gaseous, or automated decontamination of workspaces, equipment, and materials prior to removal from containment; mandatory decontamination or sterilization of all reusable instruments and materials; multi-stage personnel exit procedures incorporating surface decontamination of PPE, programmed full-body suit decontamination within exit chambers, validated doffing and suit-integrity checks, and access to personal shower facilities prior to leaving containment; controlled and documented exit sequences for personnel and materials; programmed decontamination of all items exiting the autopsy suite; defined procedures for full facility shutdown and comprehensive decontamination prior to maintenance or re-entry; coordinated communication with upstream and downstream parties to prevent inadvertent exposure during body, specimen, or material handling

^a^Summary provided for reference only; original documents should be consulted where necessary.

^b^In European/UK biosafety and regulatory terminology, pathogens are classified into Hazard Groups (HG1–4). HG3 pathogens typically correspond to work requiring BSL-3 laboratory conditions.

^c^Mpox only: Prior to removing PPE, outer gloves and any PPE with obvious contamination should be sprayed or wiped with EPA-registered disinfectant.

Earlier UK guidance from the Health and Safety Executive, Britain’s national regulator for workplace health and safety, had specified the BSL-3 infections for which a post-mortem may be carried out with transmission-based precautions [74]. The extensive 2025 RCPath guidelines now provide pathologists and mortuary staff with comprehensive recommendations for conducting such autopsies safely.

In the US, the CDC has issued two pathogen-specific protocols: an interim COVID-19 guidance for collection and submission of post-mortem specimens, last updated in 2022 and now archived, and a more recent protocol on autopsy and handling of human remains from patients with Mpox, issued in 2024. Broader recommendations on autopsy safety had previously appeared in the CDC’s 2012 “Guidelines for Safe Work Practices in Human and Animal Medical Diagnostic Laboratories” [75].

The two CDC protocols share identical PPE requirements, and both include engineering controls to limit aerosol generation as well as post-mortem containment. The COVID-19 guidance devotes an extensive section to cleaning and waste disposal, with detailed instructions for decontamination of instruments, sharps management, and surface disinfection. However, it remains narrow in its standard sampling recommendations, focusing on the respiratory tract despite the well-documented multi-organ tropism of SARS-CoV-2 [76]. The Mpox protocol, by contrast, places strong emphasis on systematic, comprehensive sampling, with an extensive list of organs to be collected but omits guidance on staff training or role definition.

Unlike the CDC, which issues governmental public health guidance, the RCPath is a professional body rather than a regulatory authority. Nevertheless, its recommendations carry considerable weight in UK practice and are developed in alignment with statutory health and safety regulations. Facility infrastructure is a core element in all three protocols: Both CDC and RCPath guidance recognize the importance of directional airflow control to limit aerosol dispersion. While the CDC mandates the use of negative-pressure rooms with at least 12 air changes per hour and local laminar airflow systems, the RCPath recommends a dedicated “high-risk suite” with whole-room downward airflow from ceiling to floor, or alternatively the use of down-draft tables, emphasizing directional control at the room or table level.

Approaches to risk assessment also diverge. The CDC COVID-19 guidance leaves the decision to professional judgment, supported by a site-specific risk assessment before any procedures are undertaken. The Mpox protocol is more restrictive: it explicitly states that autopsies should only be performed to the extent necessary to obtain “needed information,” and that examinations likely to generate aerosols or increase environmental contamination should be omitted. The RCPath framework is considerably more thorough. It requires a structured case-by-case assessment that incorporates clinical history, pathological and infection control information, and external examination of the body.

All three protocols recognize the aerosol risks of oscillating saws and prescribe engineering controls, with the RCPath guidance further recommending that in high-risk cases, the head and saw be encased in a transparent plastic bag during cranial opening. Notably, however, none of the available protocols provide recommendations on body closure following autopsy, leaving a practical aspect of post-mortem handling unaddressed.

In addition to these national guidelines, institutional HCID autopsy protocols such as the one developed at the Beijing Ditan Hospital during the 2002–2004 SARS outbreak provide further examples of high-containment autopsy procedures at the BSL-3 level [77]. While not a national standard, the Beijing protocol exemplifies the use of advanced infrastructure – including strict three-zone separation and down-draft containment tables – and represents a fully realized autopsy model for BSL-3 containment requiring HCIDs.

## Translating autopsy guidance to BSL-4 containment

The 2002–2004 SARS outbreak inspired the development of an even more advanced approach in Singapore: the development of a mobile BSL-4 autopsy unit by the Centre of Forensic Medicine of the Singapore Health Sciences Authority. Initially conceived as a contingency for future outbreaks of BSL-4 containment requiring pathogens, the unit has since been used for autopsies during the COVID-19 pandemic. Designed for rapid deployment and fully containerized, this facility meets BSL-4 containment standards and eliminates the need to transport highly infectious bodies over long distances, as the unit can be shipped by air, land, or sea. Technically, the unit consists of two 40-foot refrigerated containers: an autopsy container with a stainless-steel internal shell, changing room, pass-through decontamination chamber, and personnel shower, as well as a support container housing the suit systems, control room, and engineering subsystems [78,79].

While these infrastructural innovations that emerged during past outbreaks demonstrate that high-containment autopsies are technically feasible, they cannot substitute for clear frameworks to standardize BSL-4 autopsy practice in the context of HCIDs.

Building on the procedural principles established in BSL-3 autopsy guidance, but recognizing the fundamentally different containment paradigm at BSL-4, we propose an approach that adapts and escalates these elements under conditions of maximal biocontainment (Table 1, column 4). This approach does not seek to broaden routine autopsy practice, but rather to serve as a starting point for discussion on how to define the conditions under which invasive post-mortem procedures may be considered exceptional yet feasible when justified by diagnostic, public health, or substantial scientific need, and when institutional authorization, facility readiness, and personnel competence are assured.

**Figure 1.**
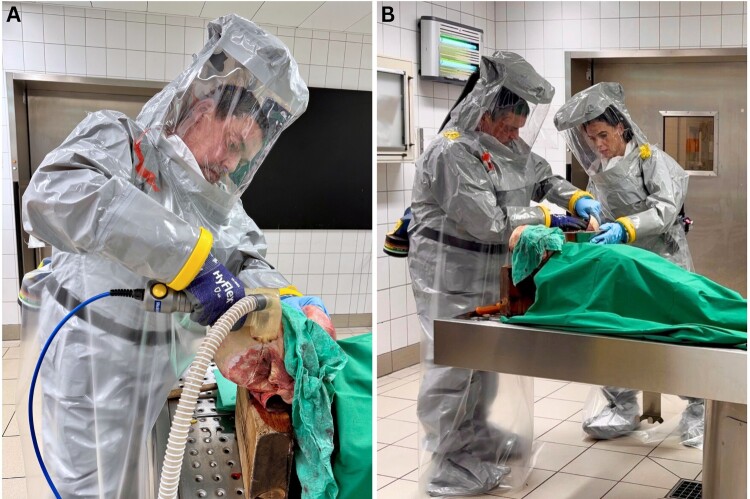
Autopsy training on a non-infectious corpse simulating BSL-4 conditions at the Institute of Legal Medicine Hamburg-Eppendorf. (a) For high-risk activities such as saw handling, the use of cut-resistant gloves (disposed of after use) worn over at least two pairs of nitrile gloves is recommended. (b) Fully encapsulating positive-pressure protective suits are required for all operators involved during BSL-4 autopsies. For reasons of dignity and to prevent contamination, the body was covered. All identifying information was pixelated.

In contrast to BSL-3 guidance, where risk mitigation often relies on procedural limitation and avoidance of aerosol-generating steps, the BSL-4 framework shifts emphasis towards containment-based control within a fully licensed and monitored environment. Procedures associated with an increased risk of occupational injury and pathogen exposure, such as saw use, are not performed routinely and remain subject to strict justification within BSL-4 autopsy guidelines and procedure-specific standard operating procedures (SOPs); however, they may be undertaken when all containment, engineering, and operational prerequisites are met, and personnel have been appropriately trained (Figure 1). Accordingly, procedural decision-making at the BSL-4 level is governed less by the intrinsic risk of individual techniques than by the integrity of the containment system and the robustness of institutional oversight.

Consistent with this containment-based paradigm, staff organization under BSL-4 conditions emphasizes functional role differentiation, continuous supervision, and integrated engineering and emergency support, shifting responsibility from individual procedural autonomy to system-level reliability. Throughout this framework, full autopsies under BSL-4 conditions are understood as exceptional procedures rather than routine practice, yet they represent a critical investigative tool for elucidating the pathophysiology of HCIDs and for enabling systematic studies that generate evidence not obtainable through alternative approaches.

## Conclusion and outlook

Autopsies remain an indispensable tool for understanding the pathogenesis of emerging infectious diseases, especially those caused by high-consequence pathogens such as VHFs. While the COVID-19 pandemic helped to re-establish the relevance of post-mortem examinations and spurred the development of new institutional and national frameworks, major gaps persist in the procedural governance of autopsies under BSL-4 conditions. International and national protocols focus primarily on post-mortem handling and disposal of the deceased, offering only limited provisions for specimen collection and explicitly discouraging full autopsies. In contrast, some BSL-3 autopsy protocols provide structured, implementable procedures, demonstrating that safe autopsy practice under elevated biosafety conditions is both feasible and already well-regulated in certain settings.

Innovative practices such as robotic biopsy systems and mobile autopsy units offer promising avenues for addressing biosafety and logistical challenges, but remain isolated examples rather than components of a standardized international or real-life approach. Likewise, autopsy alternatives such as Virtopsy and MITS can augment but not replace full post-mortem investigations in high-consequence infection scenarios.

Moving forward, there is an urgent need to close the regulatory and procedural gaps for BSL-4 autopsies. Documented cases of fatal infections have demonstrated the inherent risks of such procedures and underscore the necessity of robust guidelines. This includes the development of detailed, internationally harmonized protocols that define minimum infrastructure, training standards, and operational procedures for post-mortem investigations involving HCID pathogens and to roll them out at least in distinct centres of excellence. Particular attention should also be paid to integrating cultural considerations, improving global access to high-containment facilities, and enabling collaborative research through standardized sampling and data sharing frameworks.

At the same time, it must be acknowledged that BSL-4 facilities are associated with substantial costs, both for establishment and long-term maintenance, rendering such infrastructure largely inaccessible for many low- and middle-income countries. In this context, international collaboration will be essential to facilitate safe and equitable access to BSL-4 autopsy capabilities, including mechanisms for shared expertise, capacity building, and cross-border deployment of resources. Modular or mobile high-containment autopsy units, such as container-based facilities, may represent a pragmatic solution to enable post-mortem investigations in outbreak-affected regions while maintaining appropriate biosafety standards.

The approach to BSL-4 autopsy practice described in this work may serve as a structured starting point for broader discussion and the future development of evidence-informed, internationally harmonized guidelines. As future outbreaks are inevitable, establishing a comprehensive and globally coordinated autopsy infrastructure – both in terms of biosafety and scientific methodology – should become a central component of pandemic preparedness. Beyond determining causes of death, high-quality post-mortem investigations can yield critical insights into disease mechanisms and pathophysiology, inform clinical management strategies, and support the development of effective public health responses.

## Supplementary Material

Supplemental Table S1.docx
