# Supplementary material for: Autopsy practices for high-consequence infectious diseases: global guidelines, alternatives, and the BSL-4 gap
Source: Emerg Microbes Infect. 2026 May 23;15(1):2678656. doi: 10.1080/22221751.2026.2678656 (PMC13250869; doi:10.1080/22221751.2026.2678656)
Supplement: Supplemental Table S1.docx [file TEMI_A_2678656_SM7301.docx]

# **Supplemental Table S1: Comparison of published BSL‑4 Postmortem Care Guidelines^[[1]](#endnote-1)^**

| **Aspect** | **USA (CDC)** [57,58] | **WHO** [59–61] | **Germany (RKI)** [62–64] | **UK** [65–67] | **Australia** [68] |
| --- | --- | --- | --- | --- | --- |
| **Pathogen Scope** | Ebola, Marburg,  Lassa, Crimean–Congo hemorrhagic fever,  South American VHFs^[[2]](#endnote-2)^ | Ebola, Marburg | Ebola, Marburg | all VHFs | Ebola |
| **PPE** | **Respiratory, head, face protection^[[3]](#endnote-3)^:**  N95 respirator (or higher) in combination with surgical hood extending to shoulders and face shield, or PAPR^[[4]](#endnote-4)^ | **Respiratory, head, face protection:**  goggles or face shield,  respirator (FFP2 or N95) or PAPR | **Respiratory, head, face protection^[[5]](#endnote-5)^:**  CE category II non-fogging goggles, hood as applicable,  FFP3 half-mask with exhalation valve or TH3P blower-supported respiratory protection | **Respiratory, head, face protection^[[6]](#endnote-6)^:**  full-face visor, hood covering head, neck, and shoulders made of fluid-resistant, moisture-vapor permeable material with bound seams, FFP3/PAPR | **Respiratory, head, face protection:**  face shield or goggles, fluid-resistant P2 or N95 mask/respirator or PAPR, head/neck cover |
|  | **Hand protection:**  double disposable gloves with extended cuffs (at least the outer gloves) | **Hand protection:**  double gloves (nitrile preferred) with long cuff outer gloves | **Hand protection:**  three pairs of liquid-tight gloves (preferably middle pair with elongated cuff and outer pair with indicator) | **Hand protection:**  three pairs of disposable gloves (inner: short, nitrile, under gown cuff; middle: long, nitrile, over cuff; outer: standard or heavy duty, activity-dependent) | **Hand protection:**  double disposable gloves with the outer pair covering the gown’s cuff (preferably nitrile) |
|  | **Body protection:**  impermeable gown extending to at least mid-calf or disposable impermeable coverall; if coverall has exposed zipper: apron that covers the torso to the level of the mid-calf | **Body protection:**  disposable gown or coverall made of fabric tested for resistance to penetration by blood/body fluids/blood-borne pathogens, disposable, waterproof apron | **Body protection:**  cat. III type 3B disposable protective suits with hood and integrated bootees (with ankle cuff and drip guard), plastic apron recommended | **Body protection:**  rear-fastening, reinforced surgical gown made of fluid-resistant material; wide, extra-long, medium thickness plastic apron | **Body protection:**  surgical scrub or work uniform, long-sleeved fluid-resistant gown to the mid-calf (coveralls not recommended due to risk of heat stress/ contamination during removal), plastic apron |
|  | **Foot protection:**  single-use boot covers extending to at least mid-calf, single use ankle-high shoe covers (recommended) | **Foot protection:**  waterproof boots or (if not available) shoes with puncture-resistant soles and disposable overshoes | **Foot protection:**  S5 safety shoes or S5 rubber boots | **Foot protection:**  reusable surgical wellington boots, long enough to allow gown/apron overlap boot by 10-15cm | **Foot protection:**  enclosed, fluid- and sharps-resistant footwear, and single-use fluid-repellent boot covers to mid-calf |
| **Postmortem Containment** | **Implantable medical devices:**  no removal of devices or equipment | **Implantable medical devices:**  not mentioned | **Implantable medical devices:**  no removal | **Implantable medical devices:**  no removal | **Implantable medical devices:**  no removal of devices or equipment |
|  | **Body bagging:**  three pre-opened cremation-compatible body bags placed on a gurney: bag 1: chlorine-free vinyl (≥152µm, heat-sealed seams, zipper closure); bag 2: leakproof, fluid-impervious chlorine-free material, closed using thermal sealer with double and diagonal heat-seals; bag 3: laminated chlorine-free vinyl (≥457µm, heat-sealed seams, reinforced handles, zipper closure), all bags externally disinfected with EPA-registered hospital disinfectant | **Body bagging:**  vinyl body bag (≥ 400µm), should be able to hold 100-125kg, at least 4 handles, provide full containment of blood borne pathogens; external disinfection of bag (e.g., 0.5% chlorine solution); healthcare setting: double body bags, sealed, leak-proof bags, placed inside coffin and buried promptly; village setting: single body bag inside home, place in coffin outside, allow family to close coffin if they wish (using gloves) | **Body bagging:**  body covered with absorbent powder (fluid-binding, disinfectant effect), wrapped in two cloths soaked in 10% formalin solution, enclosed in two well-sealed, fluid-resistant plastic body bags (e.g., inner silver-grey, outer white), closures sealed with liquid-tight tape, each bag disinfected externally with RKI-listed disinfectant before casketing; bag placed in coffin with ≥5cm absorbent layer (sawdust, fleece, etc.), sealed, marked as highly infectious | **Body bagging:**  double body bag, sealed and disinfected,  absorbent wraps between each bag, bag sealed and disinfected with ~0.1% available chlorine equivalent, labelled high risk of infection and placed in a robust coffin with sealed joints; in case of death in bed isolator, body should be removed according to the local operational policy | **Body bagging:**  surgical mask on the deceased triple body bag (sealable, leak-proof, ≥150µm, selected in consultation with the receiving mortuary), outer body bag surface with low-concentration hypochlorite solution, labeled with name of deceased and marked as Ebola case, placed in hermetically sealed casket |
|  | **Transportation:** minimize transportation and coordinate with authorities; avoid transporting non-cremated remains via aircraft | **Transportation:** village setting: family may carry coffin (with gloves); coffin placed on rear platform of burial team car; family may ride on platform to cemetery | **Transportation:** transfer to nearest crematory by trained personnel, under official supervision | **Transportation:** transportation of bodies out of the country not recommended; ashes may be transported safely | **Transportation:** no special precautions when transporting hermetically sealed casket |
| **Funeral and Ritual Practices** | no embalming,  no washing,  no viewing,  cremated remains can be provided to family | no embalming,  no washing,  shrouding permitted, safe-burial recommendations may need to be adapted to consider cultural and religious concerns | no embalming or other manipulations of body, no viewing | no embalming,  no washing,  no dressing,  no religious or ritual preparation of body, local customs permitted within safety limits | no embalming,  no washing,  viewing (for identification purposes) possible from separate room through a window or via camera |
| **Autopsy Policy** | Autopsies not permitted unless absolutely necessary; if so, state health department and CDC need to be consulted regarding necessary precautions | Autopsies should be limited to essential evaluations and performed by trained personnel; alternatives (e.g., needle biopsies) should be considered | Autopsies not recommended for Ebola [62]; in general for cases with HCID agents, autopsies only under BSL-3/-4 conditions (e.g., treatment centres with special isolation wards) or mobile isolation units with HEPA filtration [63] | Post-mortem examination on VHF deceased should not be performed | Autopsies should be avoided; if directed by coroner and following discussion with public health unit, only by trained senior staff in physically separate room, precautions as for surgical procedures on Ebola patient |
| **Final Disposition of Remains** | Cremation; if not possible due to safety concerns (e.g., implanted devices): burial in standard metal caskets or other comparable burial methods | Cremation or prompt burial under trained team; no burial should begin until family agreement has been obtained. | Cremation preferred over burial; if cremation is refused by relatives, public health authority decides in accordance with IfSG | Cremation or burial in coffin with sealed joints (if medical devices present); UKSHA should be notified | Cremation (special regulations for jurisdictions requiring prior viewing by government medical officer; labelling for pacemaker presence) or burial in sealed coffin |

1. Summary provided for reference only; original documents should be consulted where necessary [↑](#endnote-ref-1)
2. those caused by Junin, Machupo, Chapare, Guanarito and Sabia viruses [↑](#endnote-ref-2)
3. no specific PPE details in CDC VHF postmortem document, but general referral to [58] [↑](#endnote-ref-3)
4. N95 (USA), FFP2 (EU), and P2 (Australia/New Zealand) respirators provide broadly comparable protection (≥94–95% filtration). FFP3 offers higher efficiency (≥99%) and is broadly comparable to N99. PAPRs (powered air-purifying respirators, including TH3P systems under EU standards) deliver filtered air under positive pressure and generally provide greater protection when properly used. [↑](#endnote-ref-4)
5. no specific PPE details in RKI Ebola/Marburg document, but general referral to PPE guidelines in resolution for activities outside of special isolation units involving the care of patients infected with highly pathogenic organisms or suspected of having a disease [64] [↑](#endnote-ref-5)
6. no specific PPE guidance for postmortem care in UKSHA document, but general referral to [66,67] [↑](#endnote-ref-6)
